# The Cognitive Walkthrough for Implementation Strategies (CWIS): a pragmatic method for assessing implementation strategy usability

**DOI:** 10.1186/s43058-021-00183-0

**Published:** 2021-07-17

**Authors:** Aaron R. Lyon, Jessica Coifman, Heather Cook, Erin McRee, Freda F. Liu, Kristy Ludwig, Shannon Dorsey, Kelly Koerner, Sean A. Munson, Elizabeth McCauley

**Affiliations:** 1 Department of Psychiatry and Behavioral Sciences, University of Washington, 6200 NE 74th Street, Suite 100, Seattle, WA 98115 USA; 2 Department of Psychology, University of Washington, 119A Guthrie Hall, Seattle, WA 98195 USA; 3 Evidence Based Practice Institute, Inc., 929 K Street, Washougal, WA 98671 USA; 4 Department of Human Centered Design and Engineering, University of Washington, 428 Sieg Building, Seattle, WA 98195 USA

**Keywords:** Implementation strategies, Human-centered design, Usability, Cognitive walkthrough

## Abstract

**Background:**

Implementation strategies have flourished in an effort to increase integration of research evidence into clinical practice. Most strategies are complex, socially mediated processes. Many are complicated, expensive, and ultimately impractical to deliver in real-world settings. The field lacks methods to assess the extent to which strategies are usable and aligned with the needs and constraints of the individuals and contexts who will deliver or receive them. Drawn from the field of human-centered design, cognitive walkthroughs are an efficient assessment method with potential to identify aspects of strategies that may inhibit their usability and, ultimately, effectiveness. This article presents a novel walkthrough methodology for evaluating strategy usability as well as an example application to a post-training consultation strategy to support school mental health clinicians to adopt measurement-based care.

**Method:**

The Cognitive Walkthrough for Implementation Strategies (CWIS) is a pragmatic, mixed-methods approach for evaluating complex, socially mediated implementation strategies. CWIS includes six steps: (1) determine preconditions; (2) hierarchical task analysis; (3) task prioritization; (4) convert tasks to scenarios; (5) pragmatic group testing; and (6) usability issue identification, classification, and prioritization. A facilitator conducted two group testing sessions with clinician users (*N* = 10), guiding participants through 6 scenarios and 11 associated subtasks. Clinicians reported their anticipated likelihood of completing each subtask and provided qualitative justifications during group discussion. Following the walkthrough sessions, users completed an adapted quantitative assessment of strategy usability.

**Results:**

Average anticipated success ratings indicated substantial variability across participants and subtasks. Usability ratings (scale 0–100) of the consultation protocol averaged 71.3 (*SD* = 10.6). Twenty-one usability problems were identified via qualitative content analysis with consensus coding, and classified by severity and problem type. High-severity problems included potential misalignment between consultation and clinical service timelines as well as digressions during consultation processes.

**Conclusions:**

CWIS quantitative usability ratings indicated that the consultation protocol was at the low end of the “acceptable” range (based on norms from the unadapted scale). Collectively, the 21 resulting usability issues explained the quantitative usability data and provided specific direction for usability enhancements. The current study provides preliminary evidence for the utility of CWIS to assess strategy usability and generate a blueprint for redesign.

**Supplementary Information:**

The online version contains supplementary material available at 10.1186/s43058-021-00183-0.

Contributions to the literature

- Implementation strategies are often complex, but the field lacks methods to assess the extent to which strategies are usable and aligned with the needs and constraints of the individuals and contexts who will deliver or receive them.
- The Cognitive Walkthrough for Implementation Strategies (CWIS) is a novel method for assessing strategy usability, filling a critical gap in the implementation literature and informing implementation strategy tailoring, adaptation, and redesign processes.
- Findings from the current application indicate that CWIS may have utility for identifying implementation strategy usability problems and informing contextually appropriate strategy redesign.

## Background

The past two decades have brought growing realization that research evidence—often codified in evidence-based interventions and assessments—is used infrequently, inconsistently, or inadequately in standard clinical care across numerous domains [1, 2]. Implementation strategies are techniques used to enhance the adoption, implementation, and sustainment of new practices and may be discrete (i.e., involving single actions or processes) or multifaceted (i.e., combining two or more discrete strategies) [3]. These strategies are now rapidly proliferating, with multiple compilations identified across a variety of health service delivery sectors [4, 5].

The complexity of implementation strategies varies widely, but most strategies are socially mediated processes that rely, in large part, on interactions among providers, implementation intermediaries/practitioners, or researchers [6]. Multifaceted and multi-level strategies are increasingly common, some of which are intended to be delivered over one or more years [7–9]. Implementation strategy complexity has been fueled, in part, by assumptions that multi-component and multi-level strategies may be more effective in promoting positive implementation outcomes [10, 11]. However, this perspective has been disputed [12], and significant complexity may leave implementation strategies unwieldy, expensive, and ultimately impractical. For instance, the Availability, Responsiveness, and Continuity strategy [8] is an effective multifaceted organizational approach for improving children’s mental health services, but its year-long delivery timeline may create barriers. Although the selection and tailoring of implementation strategies to address contextual determinants has emerged as a major focus of contemporary implementation research [7, 13, 14], we lack methods to assess the extent to which strategies are usable and aligned with the specific needs and constraints of the individuals who will use them. To be maximally relevant and useful, it is important to ensure that such methods are pragmatic [15, 16], meaning that they should be efficient and low burden, feasible to conduct in real-world settings, and yield actionable information that directly informs decisions about implementation strategy design.

### Human-centered design (HCD)

Methods from the field of human-centered design (HCD) have potential to drive assessment of implementation strategy usability and ensure contextual fit in ways that meet pragmatic criteria. HCD is focused on developing compelling and intuitive products, grounded in knowledge about the people and contexts where an innovation will ultimately be deployed [17, 18]. This is accomplished using many techniques to understand user experiences, such as heuristic (i.e., principle-based) evaluation, cognitive walkthroughs, and co-creation sessions [19]. Although little work has applied HCD specifically to implementation strategies, an emerging literature has begun to discuss the potential of HCD methods, processes, and frameworks for strategy development and redesign [4, 20]. Nevertheless, despite the value of overarching frameworks, specific methods are needed surrounding data collection and synthesis to drive implementation strategy development and adaptation.

*Usability*, the extent to which a product can be used by specified individuals to achieve specified goals in a specified context [21], is a key outcome of HCD processes. Usability is also a critical factor driving the adoption and delivery of new innovations, including implementation strategies [19, 22, 23]. Although potentially overlapping with perceptual implementation outcomes such as acceptability, feasibility, and appropriateness, usability can be distinguished as a characteristic of an innovation (e.g., a complex implementation strategy) and an “upstream” implementation determinant. Indeed, recent literature has described the overlap among these four constructs based on their level of contextual dependence, and identified usability as the most contextually independent [24]. Nevertheless, a lack of studies exploring usability in implementation has impeded examination of these relationships [25].

A major advantage of HCD is that it emphasizes rapid and efficient information collection to assess the degree to which a product is compelling and usable. A second advantage is to identify *usability problems*, or aspects of an innovation and/or a demand on the user which make it unpleasant, inefficient, onerous, or impossible for the user to achieve their goals in typical usage situations [26]. Existing methods such as concept mapping, group model building, conjoint analysis, and intervention mapping have great potential for strategy tailoring [7], but do not address the core issue of strategy usability.

### Cognitive walkthroughs

*Cognitive walkthroughs* are a low-cost assessment method commonly used in HCD usability evaluations, with the potential to identify aspects of complex implementation strategies that may inhibit their use (and ultimately their effectiveness) in community contexts. Walkthroughs may be used in conjunction with other strategy selection or tailoring approaches (e.g., concept mapping). Most typically, cognitive walkthroughs are designed to simulate the cognitive behavior of users by specifically asking questions related to users’ internal cognitive models and expectations for particular scenarios and tasks [27], especially in the context of “first time use” (i.e., use without prior or significant exposure to a specific product, interface, or protocol) [28]. Many variants exist [29], and walkthroughs may be conducted either one-on-one or in a group format. Relative to individual methods, group walkthrough procedures may minimize associated costs and capitalize on opportunities for interactions among users, thus enhancing the depth and quality of the resulting data [30]. Despite their near ubiquity in much of the HCD literature, cognitive walk-throughs have not been applied to the evaluation of implementation strategies.

### Current aims

This article presents a novel, pragmatic cognitive walkthrough methodology for evaluating implementation strategy usability by identifying, organizing, and prioritizing usability issues as a component of a larger strategy redesign process. We also describe an example application of the walkthrough methodology to a single implementation strategy: post-training consultation for child and adolescent mental health clinicians working in the education sector, who had recently completed training in measurement-based care. Schools have long been the most common setting in which children and adolescents receive mental health services in the USA [31, 32]. Measurement-based care (MBC)—the systematic collection and use of patient symptom and functioning data to drive clinical decision making [33]—is a well-supported practice for improving mental healthcare delivery [34, 35]. MBC is well aligned with the school setting, but inconsistently applied by school-based mental health clinicians [36, 37]. For clarity of presentation, the example used includes just one implementation strategy (consultation), one service sector (education sector mental health), one system level/user group (clinician service providers—a primary user group for post-training consultation), and one evaluation cycle, rather than all aspects of a multifaceted implementation initiative with multiple strategy iterations. Nevertheless, the walkthrough method is designed to be generalizable across implementation strategies, settings, system levels, and users. The methodology is intended for application by implementation researchers or practitioners who seek to ensure that the strategies they employ are easy to use and useful for relevant stakeholders. As such, it is expected to be most useful during pre-implementation phases of an initiative, prior to strategy deployment.

## Methodology and case study application

### Cognitive Walkthrough for Implementation Strategies (CWIS) overview

**Fig. 1:**
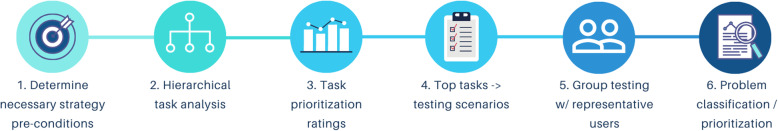
Overview of the Cognitive Walkthrough for Implementation Strategies (CWIS) methodology

The Cognitive Walkthrough for Implementation Strategies (CWIS; pronounced “swiss”) is a streamlined walkthrough method adapted to evaluate complex, socially mediated implementation strategies in healthcare. CWIS is pragmatic [38] and uses a parsimonious group-based data collection format to maximize the efficiency of information gathering. As described below, the CWIS methodology includes six steps: (1) determine preconditions; (2) hierarchical task analysis; (3) task prioritization; (4) convert tasks to scenarios; (5) pragmatic group testing; and (6) usability issue identification, classification, and prioritization (Fig. 1).

#### Example application: post-training consultation

Below, our descriptions of the CWIS steps are followed by an application to post-training, expert consultation for clinicians. Consultation involves ongoing support from one or more experts in the innovation being implemented and the implementation process [5, 39]. Given that studies consistently document initial training alone is insufficient to effect changes in professional behavior [10, 40, 41], post-consultation has become a cornerstone implementation strategy in mental health [42, 43]. In our example, CWIS was used to evaluate a brief (2–8 weeks) consultation strategy intended for school clinicians who had recently completed a self-paced online training in MBC. All clinicians worked either for school districts or community-based organizations providing individualized mental health services in elementary, middle, or high schools in a major urban area in the Pacific Northwest of the USA. The consultation strategy included (1) weekly use of an asynchronous message board to support knowledge gains and accountability, as well as (2) live, biweekly group calls to discuss cases, solidify skills, and promote the application of MBC practices.

### Step 1: Determine preconditions for the implementation strategy

Preconditions reflect the situations under which an implementation strategy is likely to be indicated or effective [44]. In CWIS, articulation of preconditions (e.g., characteristics of the appropriate initiatives, settings, individuals, etc.) by individuals with detailed knowledge of the strategy (e.g., strategy developers or intermediaries) is necessary to ensure a valid usability test. Explicit identification of end users is a key aspect of precondition articulation, a hallmark of HCD processes [45], and critical if product developers are to avoid inadvertently basing designs on individuals like themselves [46, 47]. In CWIS, if preconditions for implementation strategies are not met, the scenarios or users with which the strategy may be applied in subsequent steps will be non-representative of its intended application. For instance, the strategy, “change accreditation or membership requirements” [5] may require clinicians or organizations who are active members of relevant professional guilds as a precondition. Context (e.g., service sector) is also relevant when articulating preconditions, as different settings may influence users’ experiences of implementation strategy usability.

#### Example application

When applied to post-training consultation for MBC, the research team identified individual-level preconditions that made clinicians appropriate candidates to receive the consultation strategy. These included that clinicians provided mental health services in the education sector for some or all of their professional deployment; had expressed (by way of their participation) an interest in adopting MBC practices; and had previously completed the online, self-paced training in MBC practices that the consultation model was designed to support. Detailed personas (i.e., research-based profiles of hypothetical users and use case situations [48];) were developed to reflect identified target users.

### Step 2: Hierarchical implementation strategy task analysis

Hierarchical task analysis includes identifying all tasks and subtasks that have independent meaning and collectively compose the implementation strategy [49]. Tasks may be behavioral/physical (e.g., taking notes; speaking) or cognitive (e.g., prioritizing cases) [50, 51]. Cognitive tasks are groups of related mental activities directed toward a goal [52]. These activities are often unobservable, but are frequently relevant to the decision making and problem-solving activities that are central to many implementation strategies. In CWIS, tasks, subtasks, and task sequences (including those that are behavioral and/or cognitive) are articulated by individuals with knowledge of the strategy by asking themselves a series of reflective questions: First, for each articulated larger task or task category, asking “how?” can facilitate subtask identification. Second, asking “why?” for each task elicits information about how activities fit into a wider context or grouping. Third, asking “what happens before?” and/or “what happens after?” can allow aspects of task temporality and sequencing to emerge. All tasks identified in Step 2 can be represented either as a table or as a flow chart.

#### Example application

Tasks in the existing MBC consultation model and tested in the CWIS study were originally informed by the core consultation functions articulated by Nadeem et al. [39] (including continued training, problem-solving, engagement, case applications, accountability, adaptation, mastery skill building, and sustainment planning). Members of the CWIS project team with expertise in clinical consultation procedures identified the tasks and subtasks in the model via an iterative and consensus-driven process that involved task generation, review, and revision. In this process, a task analysis of the protocol was completed using the three questions described above. Tasks were placed in three categories, depending on whether they related to live consultation calls, the asynchronous message board, or work between consultation sessions. A list of hierarchically organized tasks was distributed to the rest of the consultation protocol developers for review and feedback. The first author then revised the task list and distributed it a second time to confirm that all relevant tasks had been identified. A number of tasks were added or combined through this process to produce the final set of 24 unique tasks for further review and prioritization in Step 3 (Table 1).

**Table 1:** Prioritization of consultation tasks

Task	Importance mean rating	Likelihood of error mean rating	Prioritization
Access digital materials (obtain internet access, access/navigate modules or message board	5.0	4.0	Selected for testing
Present 1st MBC case (succinctly summarize case features/MBC plan, select assessment instrument OR describe results of initial assessment, describe rationale for ongoing assessments, articulate what a positive response would look like OR describe results of ongoing assessments, describe next steps for MBC with the case)	5.0	4.0	Selected for testing
Articulate possible barriers/concerns with MBC for identified cases (identify barriers, articulate likelihood of each barrier, problem-solve barrier with consultant: generate solutions, select a solution to apply)	4.8	3.5	Selected for testing
Plan for maintenance of behavior change (articulate additional barriers to MBC, problem-solve barriers with consultation group: generate solutions, select a solution to apply)	4.5	4.0	Selected for testing
Login to message board (access user name/password info, type user name/password successfully)	4.5	3.0	Deprioritized (entirely digital process)
Revise planned MBC steps with case based on feedback/discussion (change monitoring target for MBC cases: revisit goals/select alternative targets/articulate plan to present alternative targets to case, select alternative cases for MBC)	4.0	3.3	Selected for testing
Navigate to message board from 1+ location	4.0	3.3	Deprioritized (entirely digital process)
Articulate questions/problems encountered RE: MBC skill application (identify specific MBC step where assistance is needed, respond to consultant questions clarifying details about skill application: what have they tried/what are they trying to achieve/what has been problematic, *role play MBC conversation	4.0	2.5	
2nd or 3rd case presentation (succinctly summarize case features/MBC plan, select assessment instrument OR describe results of initial assessment, describe rationale for ongoing assessments, articulate what a positive response would look like OR describe results of ongoing assessments, describe next steps for MBC with the case)	3.8	3.0	
Navigate to appropriate discussion thread	3.8	3.0	
Read trainee and consultant posts within a thread	3.8	2.8	
Discuss non-completion of practice activity (generate reasons, problem solve: generate solutions, select solution to apply)	3.5	3.3	
Complete values activity (articulate professional values, articulate barriers, articulate if/then plan linking barriers to correction actions)	3.5	3.3	
Post practice activity results	3.3	3.7	
Schedule make-up calls	3.3	3.3	
Identify/post cases to serve as MBC targets (review cases to identify those appropriate for MBC, post succinct case examples on message board)	3.3	2.3	
Give Introduction (wait turn/speak at appropriate time, present info, report practice activity, summarize practice activity)	3.3	2.0	
Record new practice/homework assignment to be posted to message board	3.0	2.8	
Other practice/homework activities	2.8	2.8	
Provide feedback on others’ posts/MBC application questions (identify relevant BOLT map steps)	2.5	3.0	
Email consultants with questions	2.5	2.0	
Revise an existing MBC script to tailor to personal style/student population	2.5	1.8	
Provide constructive feedback to other trainees (wait turn/speak at appropriate time, describe ideas about MBC application)	2.3	2.8	
Document questions/concerns as they arise	1.8	2.3	

*MBC* measurement-based care

### Step 3: Task prioritization ratings

Owing to their complexity, it is rarely feasible to conduct a usability evaluation that includes the full range of tasks contained within an implementation strategy. In CWIS, tasks are prioritized for testing based on (1) the anticipated likelihood that users might encounter issues or errors when completing a task, and (2) the criticality or importance of completing the task correctly. Separate Likert-style ratings for each of these two dimensions are collected, ranging from “1” (extremely unlikely to make errors/unimportant) to “5” (extremely likely to make errors/extremely important). These ratings should be completed by individuals who have expertise in the implementation strategy, the context or population with which it will be applied, or both. Tasks are then selected and prioritized based first on importance and then on error likelihood. CWIS does not specify cutoffs for task selection, as such decisions should be made in the context of the resources and information available to the research team.

#### Example application

Tasks identified in Step 2 were reviewed and rated by four members of the research team with experience in post-training consultation and MBC. Mean importance/criticality and error likelihood ratings were calculated across respondents (Table 1). Across tasks, the two ratings were correlated at *r* = 0.71. Top-rated tasks (i.e., those with high ratings on both importance and error likelihood) were selected for testing and scenario development (see below). One highly rated task (“Log into message board”) was deprioritized since it was a fully digital process and could be readily addressed in a more traditional usability evaluation. In all, Step 3 resulted in five consultation tasks being identified for testing in the CWIS process.

### Step 4: Convert top tasks to testing scenarios

Task-based, scenario-driven usability evaluations are a hallmark of HCD processes. Once the top tasks (approximately 4–6) have been identified, they need to be represented in an accessible format for presentation and testing in cognitive walkthroughs by the research team. In CWIS, tasks from Step 3 are used to develop overarching scenarios that provide important background information and contextualize the tasks. Scenarios are generally role-specific, so the target of an implementation strategy (e.g., clinicians) might be presented with a different set of scenarios and tasks than the deliverer of an implementation strategy (e.g., expert consultants). CWIS scenarios provide contextual background information on timing (e.g., “it is the first meeting of the implementation team”), information available (e.g., “you have been told by your organization that you should begin using [clinical practice]”), or objectives (e.g., “you are attempting to modify your practice to incorporate a new innovation”). Tasks are sometimes expanded or divided into more discrete subtasks at this stage. Some scenarios might contain a single subtask while other scenarios might have multiple subtasks. Regardless, each scenario presented in CWIS should include the following components to ensure clear communication to participants: (1) a brief written description of the scenario and subtasks, (2) a script for a facilitator to use when introducing each subtask, and (3) an image or visual cue that represents the scenario and can quickly communicate the subtasks’ intent.

#### Example application

**Fig. 2:**
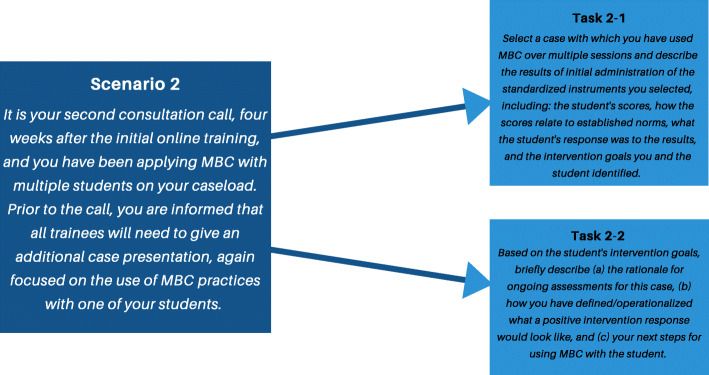
Example CWIS scenario and subtasks

Based on the prioritized tasks, the research team generated six scenarios for CWIS testing. These scenarios reflected common situations that users would be likely to encounter when participating in consultation. Each scenario contained 1–3 specific subtasks. Figure 2 displays an example scenario and its subtasks whereas Additional file 1 contains all scenarios and subtasks.

### Step 5: Group testing with representative users

In Step 5, the testing materials (Step 4) are presented to small groups of individuals (i.e., 4–6) who represent the user characteristics identified in Step 1. CWIS’ pragmatism is driven, in part, by its efficient use of user participants. Because HCD typically relies on purposive sampling of representative users, it is common to test with as few as five to seven individuals per user group. Individuals recruited reflect primary user groups (Step 1) or the core individuals who are expected to use a strategy or product [45, 53]. The primary users of implementation strategies often include both the targets of those strategies and the implementation practitioners who deliver them. For instance, testing components of a leadership-focused implementation strategy (e.g., Leadership and Organizational Change for Implementation [54];) could include representative leaders from the organizations in which the strategy is likely to be applied as well as leadership coaches from the implementation team. Regardless, it is advantageous to construct testing groups that reflect single user types to allow for targeted understanding of their needs. In addition to primary users, secondary users (i.e., individuals whose needs may be accommodated as long as they do not interfere with strategy’s ability to meet the needs of primary users) may also be specified.

CWIS sessions are led by a facilitator and involve presentation of a scenario/subtask, quantitative ratings, and open-ended discussion, with notes taken by a dedicated scribe. CWIS uses note takers instead of transcribed audio recordings to help ensure pragmatism and efficiency. First, each scenario is presented in turn to the group, followed by its specific subtasks. For each subtask, participants reflect on the activity, have an opportunity to ask clarifying questions, and then respond to three items about the extent to which they anticipate being able to (1) know what to do (i.e., discovering that the correct action is an option), (2) complete the subtask correctly (i.e., performing the correct action or response), and (3) learn that they have performed the task subtask correctly (i.e., receiving sufficient feedback to understand that they have performed the right action). They record these ratings using a 1–4 scale independently on a rating form (Additional file 2), the primary function of which is to provide participants with a concrete structure for considering each task and ultimately facilitate usability issues identification (Step 6). Next, participants sequentially provide verbal justifications or “failure/success stories,” which reveal the assumptions underlying their rating choices [29]. Any anticipated problems that arise are noted as well as any assumptions made by the participants surrounding the strategy, its objectives, or the sequence of activities. Finally, having heard each other’s justifications for their ratings, the participants engage in additional open-ended discussion about the subtask and what might interfere with or facilitate its successful completion. During this discussion, note takers attend specifically to additional comments about usability issues for subsequent classification and prioritization.

At the conclusion of a CWIS session, participants complete a quantitative measure designed to assess the overall usability of the strategy. For CWIS, our research team adapted the widely used 10-item System Usability Scale [55, 56]. The resulting Implementation Strategy Usability Scale (ISUS; Additional file 3) is CWIS’s default instrument for assessing overall usability and efficiently comparing usability across different strategies or iterations of the same strategy.

#### Example application

Potential primary users included both clinicians and MBC expert consultants (Step 1), but only clinicians were selected for testing given the modest goals of the CWIS pilot and because the deliverers of the consultation protocol (i.e., expert consultants) were already directly involved in its development. CWIS participants (*n* = 10) were active mental health clinicians who primarily provided services in K-12 education settings and had completed a self-paced, online MBC training (see Step 1: Preconditions). Participating clinicians came from a variety of organizations (i.e., multiple school districts and school-serving agencies), were 90% female, and had been in their roles for 2–18 years. Table 2 displays all participant demographics. Human subjects approval was obtained by the University of Washington Institutional Review Board, and all participants completed standard consent processes.

**Table 2:** Clinician demographics

	*N*	%
Gender		
Male	1	10
Female	9	90
Race/ethnicity		
Aboriginal (First Nations, Metis, Inuit)	0	
Native Hawaiian or other Pacific Islander	0	
Black or African American	0	
Asian	2	20
White or Caucasian	7	70
Latino	1	10
Highest degree earned		
Master’s	10	100
Age		
25–34	4	40
35–44	5	50
45–54	0	
55–64	1	10
Years in role		
Less than 5 years	3	30
5–10 years	3	30
11–15 years	3	30
16–20 years	1	10

A facilitator (first author) conducted two CWIS sessions (including 4 and 6 clinicians, respectively), lasting approximately 90 min each, and guided each group through the six scenarios and eleven associated subtasks (Additional file 1). As detailed above, users were asked to rate each task based on their personal anticipated likelihood of success discovering the correct action, likelihood of performing that action, and likelihood that they would know about the success or failure of their action. Average success ratings for each subtask were calculated as the mean of all questions and user ratings and incorporated into a matrix cross-walking the team’s original importance ratings with the success ratings generated by users.

Next, clinicians provided open-ended rating justifications and engaged in group discussion, including describing why some subtasks were considered more difficult than others and what aspects of subtasks they found particularly confusing or difficult. Discussion was recorded by the note taker for subsequent synthesis by the research team. Note takers were project staff trained by the investigators to capture qualitative explanations given by providers for their ratings. These were recorded in as much detail as possible (often verbatim) using a structured guide that facilitated tracking which task was presented, which participant was speaking, and their specific comments. Following the walkthrough sessions, users completed the ISUS in reference to all aspects of the consultation protocol to which they had been exposed.

### Step 6: Usability issue identification, prioritization, and classification

Within CWIS, usability issues are identified, classified, and prioritized using a structured method to ensure consistency across applications. All usability issues are identified by the research team, based on the results of Step 5 testing.

#### Identification and prioritization

In CWIS, identification of usability issues occurs in accordance with recent guidance articulated by the University of Washington ALACRITY Center [4, 57] for articulating usability issues for complex psychosocial interventions and strategies. Specifically, usability issues should include (1) a brief description (i.e., a concise summary of the issue, focused on how the strategy fell short of meeting the user’s needs and its consequences), (2) severity information (i.e., how problematic or dangerous the issue is likely to be on a scale ranging from 0 [“catastrophic or dangerous”] to 4 [“subtle problem”], adapted from Dumas and Redish [58]), (3) information about scope (i.e., the number of users and/or number of components affected by an issue), and (4) indicators of its level of complexity (i.e., how straightforward it is to address [low, medium, high]). The consequences of usability issues (a component of issue descriptions) may either be explicitly stated by participants or inferred during coding. Determinations about severity and scope are informed by the extent to which usability issues were known to impact participants’ subtask success ratings (Step 5). Usability issues that are severe and broad in scope are typically the most important to address. Those that are also low in complexity may be able to be prioritized for the most immediate changes to the strategy because they may be the easiest to immediately improve [59].

#### Classification

In CWIS, all identified usability problems are classified by the research team using a consensus coding approach and a framework adapted from the enhanced cognitive walkthrough [29]. The first category includes issues associated with the *user (U)*, meaning that the problem is related to the experience or knowledge a user has been able to access (e.g., insufficient information to complete a task). Second, an implementation strategy usability problem may be due to information being *hidden (H)* or insufficiently explicit about the availability of a function or its proper use. Third, issues can arise due to *sequencing or timing (ST)*, which relates to when implementation strategy functions have to be performed in an unnatural sequence or at a discrete time that is problematic. Fourth, problems with strategy *feedback (F)* are those where the strategy gives unclear indications about what a user is doing or needs to do. Finally, *cognitive or social (CS)* issues are due to excessive demands placed on a user’s cognitive resources or social interactions. Usability issue classification is critical because it facilitates aggregation of data across projects and allows for more direct links between usability problems and potential implementation strategy redesign solutions. For instance, user issues may necessitate reconsideration of the target users or preconditions (e.g., amount of training/experience) whereas cognitive or social issues may suggest the need for simplification of a strategy component or enhanced supports (e.g., job aids) to decrease cognitive burden. Categories are not mutually exclusive, so a single usability issue may be classified into multiple categories as appropriate.

#### Example application

Using a conventional content analysis approach [60], the ratings and notes from each CWIS session were independently reviewed and analyzed by members of the research team who identified usability issues by independently identifying issues and then meeting to compare their coding, refine the list, and arrive at consensus judgments [61]. No a priori codes were specified, as all codes were derived from the data. Next, coders independently rated issue severity and complexity. Outcomes of the application of CWIS Step 6 to the MBC consultation protocol are presented in the results below.

## Results

### Task success

**Fig. 3:**
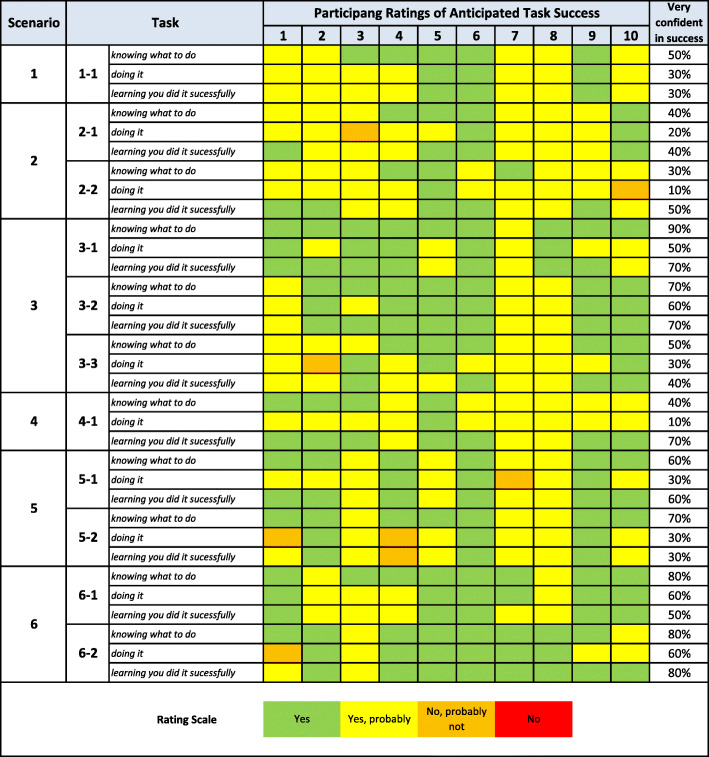
CWIS task success ratings for all subtasks and participants

Figure 3 presents a matrix of all subtasks rated by the participants, color coded based on their anticipated success (1—very small chance of success [red], 2—small chance of success [orange], 3—probable chance of success [yellow], and 4—very good chance of success [green]). The percentage of users who felt very confident in their anticipated success is highlighted in the rightmost column. Overall, ratings indicated substantial variability in anticipated success across participants and subtasks. Participants tended to rate their success knowing what to do (mean = 3.6) and learning that they did it successfully (mean = 3.53) higher than their success actually completing the subtask (mean = 3.29). Regarding specific subtasks, *linking client intervention goals to an outcome monitoring plan* received the lowest ratings.

### Overall strategy usability

ISUS ratings (scale 0–100) ranged from 57.5 to 82.5, with a mean of 71.3 (median = 72.5; *SD* = 10.6). Mean ratings for each CWIS group were similar (73.1 vs. 70.0). Based on descriptors developed for the original System Usability Scale [55], this range corresponds to descriptors between “low marginal” (1st quartile) and “excellent” (4th quartile) [62]. The mean was in the lower end of the “acceptable” range.

### Usability problems

Consensus coding yielded 21 distinct usability problems. Usability issues included potential misalignment between consultation and clinical service timelines as well as the need for tools to support real-time decision-making during consultation. Table 3 displays each of these usability problems, organized based on average severity scores completed by three members of the research team. Additional file 4 displays example excerpts from testing that supported each usability issue. Overall, usability issues ranged from the most severe at 1.33 for *Focus on barriers detracts from case presentation* to 4.00 for *Unfamiliar language in consultation model*. Usability issues rated as the most severe (1.00–2.00) demonstrated a full range of complexity levels, but were primarily high or medium complexity and, with one exception, were identified by five or more participating users. Overall, the scope of the usability issues ranged from those that affected a single user (e.g., *Case presentations exceed time allotted*) to those that were identified by seven separate users (*Unprepared to articulate monitoring targets*). Application of the adapted enhanced cognitive walkthrough categorization approach [29] indicated that approximately half of the issues could be classified within multiple categories. Nine issues were determined to be related to the *user*, three issues were related to information being *hidden,* two issues were connected to *sequencing or timing*, three issues were due to insufficient *feedback*, and eleven issues reflected excessive *cognitive or social* demands.

**Table 3:** Prioritization and categorization of usability problems

Severity rating	Complexity	Scope	Abbreviated UP	Usability problem	Problem types				
1.33	High	2	Focus on barriers detracts from case presentation	During initial case presentations, clinicians tend to focus on barriers to actually applying MBC, potentially detracting from other important topics of discussion and decreasing motivation to implement MBC (inferred).	**U**	H	ST	**F**	CS
1.67	Medium	5	Unprepared to identify solutions to barriers	When generating solutions to perceived barriers to using MBC during late-stage consultation calls, clinicians don't feel prepared to identify appropriate/insightful solutions in the moment, leaving them unsure how to proceed (stated), and discouraged or unmotivated to use MBC (inferred).	**U**	H	ST	F	CS
1.67	Medium	7	Inadequate on-site technology	Consultation calls employ videoconference technologies and equipment, but some clinicians do not have necessary hardware or technological supports, which might detract from the level of engagement or ability to participate during the calls (inferred).	U	H	ST	F	**CS**
2.00	Medium	5	Rapid assessment misaligned with available time	The consultation protocol assumes a rapid assessment and feedback process between meetings to identify treatment goals (4 weeks), which clinicians experienced as shorter than amount of time often allotted, creating a barrier to implementing MBC (stated) and/or decreased engagement with consultation (inferred).	U	H	**ST**	F	**CS**
2.00	High	5	Digressions derail barrier problem solving and engagement	When clinicians are asked to articulate and prioritize perceived barriers to applying MBC, they frequently digress, resulting in other clinicians disengaging from the call (stated), worries about describing contextual constraints of their roles (stated), and uncertainty about quality of feedback that is contingent on their ability to adequately present information (stated).	U	H	ST	**F**	CS
2.00	Low	7	Unprepared to articulate updated monitoring targets	When prompted to articulate their plan to present updated monitoring targets to the student, clinicians feel put on the spot and question the quality of the feedback they are receiving, resulting in less confidence (stated) and unwillingness to participate in the call (inferred).	U	H	ST	**F**	**CS**
2.33	Low	2	No storage for barrier solutions	When articulating possible solutions to anticipated barriers, clinicians had no clear place to store their recorded solutions, decreasing the likelihood that they would be able to access the solutions at a later time (stated).	U	**H**	ST	F	CS
2.33	High	2	Regular calls incompatible with time/availability	The consultation call model expects clinicians to attend regular/scheduled calls, which clinicians find incompatible with their time and availability, which might lead to lower participation (inferred).	U	H	ST	F	**CS**
2.67	Medium	1	Case presentations exceed time allotted	During initial case presentations, stated call expectations that presentations are brief (i.e., 1–3 min) results in clinicians potentially exceeding the time allotted (stated), which might detract from other important topics of discussion (inferred).	U	H	ST	F	**CS**
3.00	Medium	5	Unfamiliar case update structure	When providing case updates on subsequent calls, the case presentation structure (i.e., providing rationale, positive intervention response, and next steps) may be unfamiliar and a deviation from the case presentation approaches clinicians are used to, resulting in wariness and a lack of confidence (stated).	**U**	H	ST	F	**CS**
3.00	Medium	4	Duration misaligned with preferences	The consultation call model may be too brief to align with clinicians' stated preferences needing longer overall duration of consultation, potentially leading to a sense of lack of confidence and support (inferred) to effectively implement MBC**.**	U	H	ST	F	**CS**
3.00	Low	3	No continued access to resources	Upon concluding live consultation, clinicians experienced concerns over the absence of continued access to resources (guidance, training, etc.) and peer discourse, which might result in feeling a lack of support or uncertainty in how to proceed with MBC (inferred).	U	**H**	**ST**	F	CS
3.33	Low	2	Discomfort with assessments in case presentations	During initial case presentations, clinicians experience potential discomfort presenting information from MBC assessments that they have not yet mastered, leading to less confidence in implementing MBC (inferred).	**U**	H	ST	F	CS
3.33	Low	4	Confusion over MBC terminology	When presenting the results of standardized assessments during initial case presentations, clinicians experience confusion over the terminology (established norms , clinically significant) that is foundation for MBC which could lead to less confidence in using MBC practices (inferred), disengagement from the calls (inferred), and interfere with accurate score interpretation for students on their caseloads (stated).	**U**	H	ST	F	CS
3.33	Medium	4	Confidentiality concerns when reporting results	When presenting the results of standardized assessments during initial case presentations, clinicians are concerned over privacy and confidentiality (stated), which may have a negative impact on their confidence and interest in participating in group calls (inferred).	U	H	ST	F	**CS**
3.33	Medium	1	Difficulty articulating what is being measured	When reporting on individualized goals (and not on standardized measures), clinicians struggle to articulate what they are measuring, which results in hesitation (inferred) and fear of reporting incorrectly (inferred).	**U**	H	ST	F	CS
3.33	High	1	Constraints on access to school buildings/students	When discussing solutions for addressing perceived barriers during late-stage consultation calls, clinicians from outside community mental health agencies have more constraints surrounding their access to school buildings and students, which results in more limitations (and less control) surrounding the execution of their identified solutions.	U	H	ST	F	**CS**
3.33	Medium	5	Distraction from multi-tasking online during calls	When navigating online training resources (i.e. the online message board) during the call, clinicians might get distracted or struggle to follow the call discussion, which might negatively impact group discourse or engagement (inferred).	**U**	H	ST	F	**CS**
3.33	Low	2	Unaware of available follow-up supports	When identifying the most potentially impactful barriers to MBC during late-stage calls, clinicians are unaware what follow-up/feedback is available after the call is finished, resulting in a sense of lack of support (inferred).	U	**H**	ST	F	CS
4.00	Medium	2	Technological difficulties are disengaging	When having technological difficulties (login, access to resources, etc.) on the consultation call, clinicians might feel distracted and disengaged them from the call discussion and be prevented from accessing necessary resources (inferred).	**U**	H	ST	F	**CS**
4.00	Low	3	Unfamiliar language in consultation model	Overall the consultation model uses language clinicians might experience as unfamiliar, confusing, and difficult to understand, which might 'alienate' clinicians (stated) or disengage them from participation (inferred).	**U**	H	ST	F	CS

Complexity: refers to how straightforward (or not) it is to address an issue

U: User access to knowledge/experience problem

H: Hidden problem

ST: Sequence and timing problem

F: Feedback problem

CS: Cognitive or social demands problem

## Discussion

Complex and multifaceted implementation strategies are increasingly common in implementation science. The extent to which these strategies can be successfully applied by specified users to achieve their goals is a critical consideration when making decisions about implementation strategy selection and adaptation. Usability assessment has the potential to provide a key input into strategy adoption and tailoring decisions. CWIS is the first methodology developed to explicitly assess the usability of implementation strategies in healthcare.

### CWIS findings for post-training consultation

In the current example, the results of the ISUS indicated that clinician-rated usability of the original consultation protocol was at the low end of the “acceptable” range (based on existing SUS norms) and would benefit from some revision [62]. Although potentially workable for many users, this finding suggests that revisions to the strategy are likely indicated to improve ease of use and usefulness for its identified set of clinician primary users.

In addition to ratings of overall usability, CWIS walkthrough sessions revealed 21 discrete usability issues. Collectively, these issues explain the ISUS quantitative usability data and provide specific direction for usability enhancements. Most usability issues related either to whether the protocol had inaccurate expectations surrounding clinician preparation in consultation-related skills (e.g., *Unprepared to identify solutions to barriers*), various opportunities for consultation to be disrupted by participants who needed to discuss implementation barriers (e.g., *Digressions derail barrier problem solving and engagement*), the protocol’s built-in assumptions about service delivery timelines (e.g., *Rapid assessment misaligned with available time*), or digital technology-related issues (e.g., *Inadequate on-site technology*).

### Implications for strategy redesign

Much of the utility of the CWIS methodology comes from its potential to inform user-centered redesign of implementation strategies to enhance usability. Although it is beyond the scope of this paper to articulate the full strategy adaptation process (where CWIS served as a key input), the results of the current example application indicated some clear redesign directions to improve the alignment of the consultation protocol with clinician users. Focusing redesign on the highest priority problems avoids excessive changes that may not be critical. As can be seen in Table 4, which links abbreviated descriptions of the usability problems (articulated by the research team) to redesign decisions, CWIS resulted in changes to the consultation strategy in multiple ways that were unanticipated at the outset. The highest-rated usability issues (e.g., *Focus on barriers detracts from case presentation [U, F]*; *Inadequate on-site technology [CS]*) were addressed through modifications to various consultation elements, and most redesign decisions addressed multiple usability issues. For example, the project team streamlined the consultation call time (reduced to no more than 50 min) and designed brief make-up sessions (15 min) to address how *Regular calls were incompatible with time/availability (CS)* (length and duration). Assignment of a problem type classification to each usability issue further facilitated redesign. For instance, two of the three highest severity problems were categorized as issues related to the implementation strategy not being aligned with the *Users* and their knowledge base (*Focus on barriers detracts from case presentation* and *Unprepared to identify solutions to barriers*). This indicated that additional specific supports surrounding consultation-relevant skills such as case presentations and problem-solving implementation barriers were important to improving overall usability. Modifications to address these issues included developing supplemental MBC resources, providing clear examples, and creating multiple opportunities to ask questions and get support (including asynchronously).

**Table 4:** Consultation strategy redesign decisions

Usability issues	Consultation redesign
*Focus on barriers detracts from case presentation *Unprepared to identify solutions to barriers *Digressions derail barrier problem solving and engagement *Case presentations exceed time allotted *Unfamiliar case update structure	Development of Troubleshooting Guide (guidelines, examples and tips) for consultants
*Focus on barriers detracts from case presentation *Unprepared to identify solutions to barriers *Digressions derail barrier problem solving and engagement *Case presentations exceed time allotted	Clearly defined call agenda, directions and expectations for call activities and participation
*Focus on barriers detracts from case presentation *Unprepared to identify solutions to barriers *Digressions derail barrier problem solving and engagement *Unprepared to articulate updated monitoring targets *Case presentations exceed time allotted *Unfamiliar case update structure *Difficulty articulating what is being measured	Provision of examples (e.g., case presentation) via multiple formats (i.e., modeled in vivo by consultant, discussion board, handouts)
*Focus on barriers detracts from case presentation *Digressions derail barrier problem solving and engagement *Case presentations exceed time allotted *Consultation call duration too brief	Opportunity to ask overflow questions/comments to continue via asynchronous discussion board with both consultant and call group participants
*Focus on barriers detracts from case presentation *Unprepared to identify solutions to barriers *Discomfort with assessments in case presentations	Consistent use of affirmation and positive feedback during call discussion, practice activities and discussion board use
*Case presentations exceed time allotted *Unfamiliar case update structure *No continued access to resources *Unaware of available follow-up supports *Unfamiliar language in consultation model	Development of participant handbook including information on all available resources and how-tos on accessing and utilizing technology (discussion board, training resources, calls), etc.
*Distraction from multi-tasking online during calls *Technological difficulties are disengaging	Consultant screen-shares appropriate resources, materials or examples during call
*Inadequate on-site technology *Technological difficulties are disengaging	Research team available to troubleshoot any technological issues or needs during consultation calls
*Inadequate on-site technology *Distraction from multi-tasking online during calls *Technological difficulties are disengaging	Additional technical training for consultants (e.g., learning dashboard, zoom videoconference, etc.)
*Unaware of available follow-up supports *Technological difficulties are disengaging	Orientation for participants on training platform during first call (i.e., discussion board, additional resources)
*Confidentiality concerns when reporting results	Promotion of collaborative, safe environment to share via introductions, profile photos and video during calls
*Regular calls incompatible with time/availability *Duration misaligned with preferences	Participants given one of top choices (rank order) of their preferred time for consultation calls
*Regular calls incompatible with time/availability	Offered brief make-up sessions if scheduled group calls were missed (based on their availability)
*Rapid assessment misaligned with available time *Regular calls incompatible with time/availability *Duration misaligned with preferences	Reduction of consultation call time from 1–1.5 h to 50 min
*No continued access to resources *Unaware of available follow-up supports	Allowed continued access to the training modules
*Focus on barriers detracts from case presentation *No continued access to resources *Discomfort with assessments in case presentations *Unaware of available follow-up supports	Development of supplemental MBC resources and reference materials (e.g., workflow tools, standardized assessments repository, MBC tip sheets, etc.)
*Confusion over MBC terminology *Unfamiliar language in consultation model	Revised language (e.g., reduced jargon, increased readability, clear definitions, etc.) across all project materials
*Unprepared to articulate updated monitoring targets *Case presentations exceed time allotted	Broke down MBC process into manageable steps (sequence of practice activities followed by feedback) to better align with call timeline

## Limitations and future directions

The current application of CWIS has a number of limitations. First, our example application only involved applying CWIS to a single user group (clinician recipients of the strategy) and participant diversity was limited (e.g., no clinicians identified as being Black). Future applications may include more diverse professionals, including those who *deliver* implementation strategies (e.g., expert consultants, especially those unaffiliated with the study team) as well as other types of service providers and sectors (e.g., physicians in primary care). Second, the methodology was only applied to a single implementation strategy targeting the individual clinician level. Nevertheless, most implementation efforts include multiple strategies. CWIS is intended to be applicable across strategies and levels and could be similarly useful for assessing multifaceted strategies, such as organizationally focused approaches targeting system leaders [54] or complex strategies designed to simultaneously influence multiple stakeholder groups. Such applications will help to build on and broaden the preliminary evidence for CWIS generated in the current study. Third, although it is designed to approximate the hands-on experience of using an implementation strategy, CWIS still involves some level of abstraction given that participating users do not actually complete the tasks on which they are reporting. This is a common tradeoff in cognitive walkthroughs and may be one reason why walkthrough methods sometimes over-identify usability problems [63]. Future work could determine whether group-based walkthroughs produce usability results that are comparable to more—or less—intensive (and expensive) types of testing such as walkthroughs with individuals [64]. Fourth, the current study presented an example application of CWIS to demonstrate its utility, but the results described do not reflect a direct evaluation of the acceptability, feasibility, or impact of the approach relative to a comparison condition. Fifth, while CWIS is intended to be pragmatic and efficient, the extent to which all of its activities (e.g., qualitative content analysis) are feasible for real-world implementation practitioners is uncertain and should be a focus of future inquiry. In the current study, CWIS was delivered by a research team that was external to the implementing organizations. While the CWIS sessions themselves (Step 5) are relatively brief, there is inevitable preparation required (Steps 1–4) and, following the sessions, synthesis of the resulting information (Step 6), which could impact feasibility. Nevertheless, usability evaluation activities such as these are commonly applied in industry and often completed rapidly by small teams. Finally, pragmatic methods and instruments should ideally be sensitive to change [15], but the current study only involved applying CWIS at one point in the iterative development of the consultation strategy. Additional research should evaluate CWIS’s change sensitivity and ability to identify whether redesign decisions result in new usability issues or unanticipated barriers.

## Conclusion

Despite growing interest in implementation strategy selection and tailoring processes, no methods exist to evaluate usability and ensure that strategies can be successfully applied by well-specified users in their contexts of use. The current study provides preliminary evidence for the utility of CWIS to assess strategy usability and generate a blueprint for redesign. Future work should evaluate the extent to which usability, as measured by CWIS, is predictive of the fidelity with which implementation strategies (e.g., training, consultation, leadership supports) are delivered as well as their impact on implementation and health service outcomes.

## Supplementary Information

**Additional file 1.** Full scenario and task list.

**Additional file 2.** Task rating sheet.

**Additional file 3.** Implementation Strategy Usability Scale: consultation version.

**Additional file 4.** Example excerpts from CWIS session notes.

## Data Availability

Please contact the lead author for more information.

## Abbreviations

ALACRITY: Advanced Laboratory for Accelerating the Reach and Impact of Treatments for Youth and Adults with Mental Illness
CWIS: Cognitive Walkthrough for Implementation Strategies
HCD: Human-centered design
ISUS: Implementation Strategy Usability Scale
MBC: Measurement-based care
