# Supplementary material for: The Cognitive Walkthrough for Implementation Strategies (CWIS): a pragmatic method for assessing implementation strategy usability
Source: Implement Sci Commun. 2021 Jul 17;2:78. doi: 10.1186/s43058-021-00183-0 (PMC8285864; doi:10.1186/s43058-021-00183-0)
Supplement: Supplementary file 1 — Additional file 1. Full scenario and task list. [file 43058_2021_183_MOESM1_ESM.pdf]

Scenario	Task	Task Description
**1:** You have completed the four online training modules and scheduled your first post-training consultation call with an expert MBC consultant. Prior to the call, you are informed that all trainees will need to do an initial, brief case presentation in which you will describe the application of MBC to a selected student, based on the online training you have completed. At the time of the call, you have not yet applied MBC with a student, but have identified a student with which you plan to apply MBC practices.	1-1	Succinctly summarize the key features of your selected student (time limit: 1-3 min), where you and this student are in the BOLT MBC process (i.e., initial assessment; identify treatment goals; develop monitoring plan; assess, adjust & repeat; treatment completion), as well as your plan and rationale for administration of a specific standardized assessment tool. Be sure to describe the reasons why you believe the selected instrument is a good match for the identified student.
**2:** It is your second consultation call, four weeks after the initial online training, and you have been applying MBC with multiple students on your caseload. Prior to the call, you are informed that all trainees will need to give an additional case presentation, again focused on the use of MBC practices with one of your students.	2-1	Select a case with which you have used MBC over multiple sessions and describe the results of initial administration of the standardized instruments you selected, including: the student's scores, how the scores relate to established norms, what the student's response was to the results, and the intervention goals you and the student identified.
	2-2	Based on the student's intervention goals, briefly describe (a) the rationale for ongoing assessments for this case, (b) how you have defined / operationalized what a positive intervention response would look like, and (c) your next steps for using MBC with the student.
**3:** Delivery of MBC has been challenging for some of your cases due to issues such as missed sessions, abbreviated session length, and student disengagement from discussions about their MBC data. You bring these issues up during the consultation call at a time when the expert consult solicits questions or problems for the group to discuss.	3-1	Select a specific case experiencing significant barriers to MBC. Clearly articulate the perceived barriers to applying MBC and prioritize those that appear most likely to be impacting the specific case.
	3-2	With prompting from the expert consultant, review the Troubleshooting Guide (see handout) to address common problems and, with the assistance of the full consultation group, identify/generate potentially appropriate solutions. These solutions may come from the Troubleshooting Guide or additional sources.
	3-3	Select 1-2 solutions to apply to address the issue with the specified case. Articulate how you will evaluate the success of the solution and along what timeline.
**4:** You have discussed a specific case with the consultation team and have received useful feedback suggesting that it could be useful to revise your MBC plan for that student.	4-1	Based on the feedback you received, revisit your stated intervention goals and identify alternative specific monitoring targets. Articulate your plan to present the idea of shifting monitoring targets – and options for new monitoring targets – to the identified student.
**5:** During a consultation call, one of your fellow trainees states that they are unsure how to complete part of the MBC process (e.g., access relevant standardized assessment instruments; identify intervention goals). The expert consultant indicates that there are relevant materials available online (within the online training or the message board) that could be reviewed to help answer this question. You had a similar question, so you are interested in navigating to the online resources to access these materials during the phone call.	5-1	Access a web-enabled device and, as the call progresses and you continue to attend to the discussion, navigate to the online MBC training that you recently completed. Log in to the training and, within the system, navigate to the relevant resources section.
	5-2	Access a web-enabled device and, as the call progresses and you continue to attend to the discussion, navigate to the online consultation message board. Log in to the message board and navigate to the relevant discussion thread.
**6:** You are participating in your final MBC consultation session. The expert consultant indicates that it is typically helpful to anticipate and plan for future barriers that might emerge over the remainder of the year and beyond. She asks you to consider the goals you stated in the first session surrounding your use of MBC in practice and how they might intersect with known barriers, available resources, and your professional values.	6-1	Brainstorm possible barriers that might interfere with your continued use of MBC practices. Review and select the most likely or potentially impactful barriers.
	6-2	With guidance from the expert consultant, articulate possible solutions for addressing the selected/prioritized barriers using "if-then" language. Then, select 1-2 solutions per prioritized barrier and record for future reference.
