# Supplementary material for: The Cognitive Walkthrough for Implementation Strategies (CWIS): a pragmatic method for assessing implementation strategy usability
Source: Implement Sci Commun. 2021 Jul 17;2:78. doi: 10.1186/s43058-021-00183-0 (PMC8285864; doi:10.1186/s43058-021-00183-0)
Supplement: Supplementary file 2 — Additional file 2. Task rating sheet. [file 43058_2021_183_MOESM2_ESM.pdf]

Date: ____/____/______

CWIS Task Ratings

ID: _________

Task Rating Sheet					
**For each task, please consider the extent to which YOU will be successful…**		**No** *(very small chance of success)* 1	**No, probably not** *(small chance of success)* 2	**Yes, probably** *(probable chance of success)* 3	**Yes** *(a very good chance of success)* 4
**Task 1-1**	…discovering that the correct action is an option?	1	2	3	4
**Task 1-1**	…performing the correct action or response?	1	2	3	4
**Task 1-1**	…receiving sufficient feedback to understand that you have performed the right action or that the task was successfully completed?	1	2	3	4
**Task 2-1**	…discovering that the correct action is an option?	1	2	3	4
**Task 2-1**	…performing the correct action or response?	1	2	3	4
**Task 2-1**	…receiving sufficient feedback to understand that you have performed the right action or that the task was successfully completed?	1	2	3	4
**Task 2-2**	…discovering that the correct action is an option?	1	2	3	4
**Task 2-2**	…performing the correct action or response?	1	2	3	4
**Task 2-2**	…receiving sufficient feedback to understand that you have performed the right action or that the task was successfully completed?	1	2	3	4
**Task 3-1**	…discovering that the correct action is an option?	1	2	3	4
**Task 3-1**	…performing the correct action or response?	1	2	3	4
**Task 3-1**	…receiving sufficient feedback to understand that you have performed the right action or that the task was successfully completed?	1	2	3	4
**Task 3-2**	…discovering that the correct action is an option?	1	2	3	4
**Task 3-2**	…performing the correct action or response?	1	2	3	4
**Task 3-2**	…receiving sufficient feedback to understand that you have performed the right action or that the task was successfully completed?	1	2	3	4

Date: ____/____/______

CWIS Task Ratings

ID: _________

Task Rating Sheet					
**For each task, please consider the extent to which YOU will be successful…**		**No** *(very small chance of success)* 1	**No, probably not** *(small chance of success)* 2	**Yes, probably** *(probable chance of success)* 3	**Yes** *(a very good chance of success)* 4
**Task 3-3**	…discovering that the correct action is an option?	1	2	3	4
**Task 3-3**	…performing the correct action or response?	1	2	3	4
**Task 3-3**	…receiving sufficient feedback to understand that you have performed the right action or that the task was successfully completed?	1	2	3	4
**Task 4-1**	…discovering that the correct action is an option?	1	2	3	4
**Task 4-1**	…performing the correct action or response?	1	2	3	4
**Task 4-1**	…receiving sufficient feedback to understand that you have performed the right action or that the task was successfully completed?	1	2	3	4
**Task 5-1**	…discovering that the correct action is an option?	1	2	3	4
**Task 5-1**	…performing the correct action or response?	1	2	3	4
**Task 5-1**	…receiving sufficient feedback to understand that you have performed the right action or that the task was successfully completed?	1	2	3	4
**Task 5-2**	…discovering that the correct action is an option?	1	2	3	4
**Task 5-2**	…performing the correct action or response?	1	2	3	4
**Task 5-2**	…receiving sufficient feedback to understand that you have performed the right action or that the task was successfully completed?	1	2	3	4
**Task 6-1**	…discovering that the correct action is an option?	1	2	3	4
**Task 6-1**	…performing the correct action or response?	1	2	3	4
**Task 6-1**	…receiving sufficient feedback to understand that you have performed the right action or that the task was successfully completed?	1	2	3	4

Date: ____/____/______	**CWIS Task Ratings**	ID: ________

**Task Rating Sheet**					
**For each task, please consider the extent to which <u>YOU</u> will be successful…**		**No** *(very small chance of success)* **1**	**No, probably not** *(small chance of success)* **2**	**Yes, probably** *(probable chance of success)* **3**	**Yes** *(a very good chance of success)* **4**
**Task 6-2**	…discovering that the correct action is an option?	1	2	3	4
**Task 6-2**	…performing the correct action or response?	1	2	3	4
**Task 6-2**	…receiving sufficient feedback to understand that you have performed the right action or that the task was successfully completed?	1	2	3	4

<u>COMMENTS:</u>
