# Supplementary material for: The Cognitive Walkthrough for Implementation Strategies (CWIS): a pragmatic method for assessing implementation strategy usability
Source: Implement Sci Commun. 2021 Jul 17;2:78. doi: 10.1186/s43058-021-00183-0 (PMC8285864; doi:10.1186/s43058-021-00183-0)
Supplement: Supplementary file 3 — Additional file 3. Implementation Strategy Usability Scale: consultation version. [file 43058_2021_183_MOESM3_ESM.pdf]

Implementation Strategy Usability Scale:
Consultation Version

Please use the following scale to rate how much you agree with the statements below:	Strongly Disagree<br>1	Disagree<br>2	Neither Agree nor Disagree<br>3	Agree<br>4	Strongly Agree<br>5
1. I think that I would like to use this [consultation model] frequently	1	2	3	4	5
2. I found the [consultation model] unnecessarily complex	1	2	3	4	5
3. I thought the [consultation model] was easy to use	1	2	3	4	5
4. I think that I would need the support of a technical person to be able to use this [consultation model]	1	2	3	4	5
5. I found the various components of this [consultation model] were well integrated	1	2	3	4	5
6. I thought there was too much inconsistency in this [consultation model]	1	2	3	4	5
7. I would imagine that most people would learn to use this [consultation model] very quickly	1	2	3	4	5
8. I found the [consultation model] very cumbersome to use	1	2	3	4	5
9. I felt very confident using this [consultation model]	1	2	3	4	5
10. I needed to learn a lot of things before I could get going with this [consultation model]	1	2	3	4	5

Scoring:

- For each of the **odd** numbered questions, **subtract 1 from the score.**
- For each of the **even** numbered questions, **subtract their value from 5.**
- Take these new values which you have found, and add up the total score. Then **multiply this by 2.5.**

Adapted from the System Usability Scale:

Brooke, J. (1996). SUS-A quick and dirty usability scale. Usability evaluation in industry, 189(194), 4-7.

Aaron Lyon, PhD
University of Washington
2016
