# Supplementary material for: The Cognitive Walkthrough for Implementation Strategies (CWIS): a pragmatic method for assessing implementation strategy usability
Source: Implement Sci Commun. 2021 Jul 17;2:78. doi: 10.1186/s43058-021-00183-0 (PMC8285864; doi:10.1186/s43058-021-00183-0)
Supplement: Supplementary file 4 — Additional file 4. Example excerpts from CWIS session notes. [file 43058_2021_183_MOESM4_ESM.pdf]

Additional File 4

Example Excerpts from CWIS Session Notes

Usability problem	Example Quotes
Focus on barriers detracts from case presentation	"I'm actually better at knowing what to do than actually doing it. I think that's obviously something I would strive for but I have challenging clients that sometimes derail conversations and don't want to do an assessment or they come in with a crisis so I think following through with the correct response is more difficult than identifying what needs to happen."
Unprepared to identify solutions to barriers	"I think we'd have a hard time being insightful and thinking clearly as far as how I want to proceed."
Inadequate on-site technology	"My only concern is that not all sites might have that capability…and because that might be the reality that you don't have a webcam or videoconferencing available, so it would need to be web or phone."
Rapid assessment misaligned with available time	"4 weeks is a really quick turnaround to do multiple assessment measures. Sometimes it takes a couple of sessions to get students engaged, post crises or truancy issues. Doing it might be some curse words and some struggling."
Digressions derail barrier problem solving and engagement	"When we're on consultation calls since we're not face to face, people give way too much information about this session, these barriers, I can get lost or disengaged from it. Too much information, then we come back, it's like what are we trying to focus on? "
Unprepared to articulate updated monitoring targets	"But I think it's essentially revising your treatment plan but being able to articulate it on the call, it might not be very articulate, because I'd be thinking out loud and babbling."
No storage for barrier solutions	"Probably lose track of that paper so small chance of success."
Regular calls incompatible with time/availability	"It would be really hard for multiple providers at different organizations, just to arrange our schedule to have the same consistent time and also having crises pop up with your clients or within the school. So, I just think that would be a barrier."
Case presentations exceed time allotted	"In theory it's very quick, but in experience I think people want to give a lot of context. If people stick what they're being asked, then they'd be able to do the 1-3. I'd be able to do it, but I have found myself wanting to give more."
Unfamiliar case update structure	"I think the only thing was knowing what format I'd be expected to use when defining operationalizing the positive intervention response, so without actually know that, the confidence or probability is in question."
Duration misaligned with preferences	"I think the 6-month period is good, especially with measuring outcomes but I feel kind of opposite, coming from a larger agency we already have so many monthly team meetings, SIT team meetings and consultation with children's experts. It would be really hard for multiple providers at different organizations, just to arrange our schedule to have the same consistent time and also having crises pop up with your clients or within the school. So, I just think that would be a barrier."
No continued access to resources	"Would like to have continuing to have access to online materials. I like the idea of a message board, I'm questioning whether I'd use it. Things get busy, but it would be nice."
Discomfort with assessments in case presentations	"Very atypical. The consultations I've had have been more narrative, more stories and context. I can't recall if I've ever shared a score or standardized assessment results in consultations."
Confusion over MBC terminology	"I would stumble with the established norms. I don't think this call would talk about that – I don't know what the language is about that in the consultation call."
Confidentiality concerns when reporting results	"I guess in individual supervision I'd maybe discuss a score with my supervisor, but not in a group – for privacy and confidentiality."
Difficulty articulating what's being measured	"I think talking about barriers to treatment is a frequent topic of conversation. But I don't think I've talked about barriers to measurement, specifically."

Constraints on access to school buildings/ students	"The specifics of being community mental health versus typical school-based. The limitations we have, the availability of school, I feel like solutions are a little more limited because you don't have as much access to students on a regular basis."
Distraction from multi-tasking online during calls	"I don't know if I can attend to the conversation, navigating and being present."
Unaware of available follow up supports	"I've done consultation calls with a specific model…but when they end, you're like I think this is right, I'm kind of doing this right, but I don't know for sure. Consultation calls can be a crapshoot sometimes."
Technological difficulties are disengaging	"I'm the worst at remembering login information, like I have to find the secret place and I'd need to deal with that part."
Unfamiliar language in consultation model	"Wasn't sure if this is the way the consultant is communicating to everyone on call, it was hard for me to understand what was going on. I need more plain language. I can't speak to everyone, but for me that seemed a little alienating."
